# Neurological disorder-associated genetic variants in individuals with psychogenic nonepileptic seizures

**DOI:** 10.1038/s41598-020-72101-8

**Published:** 2020-09-16

**Authors:** Costin Leu, Jocelyn F. Bautista, Monica Sudarsanam, Lisa-Marie Niestroj, Arthur Stefanski, Lisa Ferguson, Mark J. Daly, Lara Jehi, Imad M. Najm, Robyn M. Busch, Dennis Lal

**Affiliations:** 1 Genomic Medicine Institute, Lerner Research Institute, Cleveland Clinic, Cleveland, OH 44195 USA; 2 Stanley Center for Psychiatric Research, Broad Institute of Harvard and M.I.T, Cambridge, MA 02142 USA; 3 Department of Clinical and Experimental Epilepsy, Institute of Neurology, University College London, Queen Square, London, WC1N 3BG UK; 4 Epilepsy Center, Neurological Institute, Cleveland Clinic, Cleveland, OH 44195 USA; 5 Department of Neurology, Neurological Institute, Cleveland Clinic, Cleveland, OH 44195 USA; 6 Cologne Center for Genomics (CCG), University of Cologne, Cologne, DE 50931 USA; 7 Department of Psychiatry & Psychology, Neurological Institute, Cleveland Clinic, Cleveland, OH 44195 USA; 8 Institute of Molecular Medicine Finland (FIMM), Helsinki Institute of Life Science (HiLIFE), University of Helsinki, Helsinki, Finland; 9 Analytic and Translational Genetics Unit, Massachusetts General Hospital, Boston, MA 02114 USA

**Keywords:** Disease genetics, Diagnostic markers, Neurological disorders, Psychiatric disorders, Next-generation sequencing, Genetic predisposition to disease

## Abstract

Psychogenic nonepileptic seizures (PNES) are diagnosed in approximately 30% of patients referred to tertiary care epilepsy centers. Little is known about the molecular pathology of PNES, much less about possible underlying genetic factors. We generated whole-exome sequencing and whole-genome genotyping data to identify rare, pathogenic (P) or likely pathogenic (LP) variants in 102 individuals with PNES and 448 individuals with focal (FE) or generalized (GE) epilepsy. Variants were classified for all individuals based on the ACMG-AMP 2015 guidelines. For research purposes only, we considered genes associated with neurological or psychiatric disorders as candidate genes for PNES. We observe in this first genetic investigation of PNES that six (5.88%) individuals with PNES without coexistent epilepsy carry P/LP variants (deletions at 10q11.22-q11.23, 10q23.1-q23.2, distal 16p11.2, and 17p13.3, and nonsynonymous variants in *NSD1* and *GABRA5*). Notably, the burden of P/LP variants among the individuals with PNES was similar and not significantly different to the burden observed in the individuals with FE (3.05%) or GE (1.82%) (PNES vs. FE vs. GE (3 × 2 χ^2^), *P* = 0.30; PNES vs. epilepsy (2 × 2 χ^2^), *P* = 0.14). The presence of variants in genes associated with monogenic forms of neurological and psychiatric disorders in individuals with PNES shows that genetic factors are likely to play a role in PNES or its comorbidities in a subset of individuals. Future large-scale genetic research studies are needed to further corroborate these interesting findings in PNES.

## Introduction

PNES is considered a multifactorial biopsychosocial disorder^1^, that can coexist with epilepsy^2^, and which has a broad range of comorbid neurological and psychiatric disorders. Major depression and anxiety disorders are the most common psychiatric comorbidities, both reported in ~ 50% of all individuals with PNES^3,4^. PNES, epilepsy, and psychiatric disorders cluster in families with a positive family history of psychiatric disorders in 7–22% of all individuals with PNES^5,6^ and a positive family history of epilepsy in 7–48% of all individuals with PNES^5–7^. However, the upper ranges of the estimates for a positive family history of psychiatric disorders and epilepsy are driven by the inclusion of individuals with PNES and comorbid epilepsy. Such individuals were not routinely excluded in the majority of all PNES studies.

Recent results indicate that some genetic risk factors are shared between common neurological and psychiatric disorders (including epilepsy, depression, anxiety, and neuroticism)^8,9^ and may explain part of the observed phenotypic overlap^10^. Widespread pleiotropy for genes associated with Mendelian forms of neurological and psychiatric disorders suggests that these disorders are the response of a complex neurologic network, which is altered in several domains of function. For example, individuals affected by pathogenic variants in well-established epilepsy genes usually have additional neurological or psychiatric comorbidities^11^. While recent success in genetic studies has led to the identification of disease-associated genetic factors for virtually all disorders comorbid with PNES (i.e., anxiety, bipolar disorder, depression, epilepsy, intellectual disability, migraine, mood disorders, personality disorders, post-traumatic stress disorder, and schizophrenia), a genetic basis of PNES has been speculated^12^, but never formally investigated.

**Figure 1:**
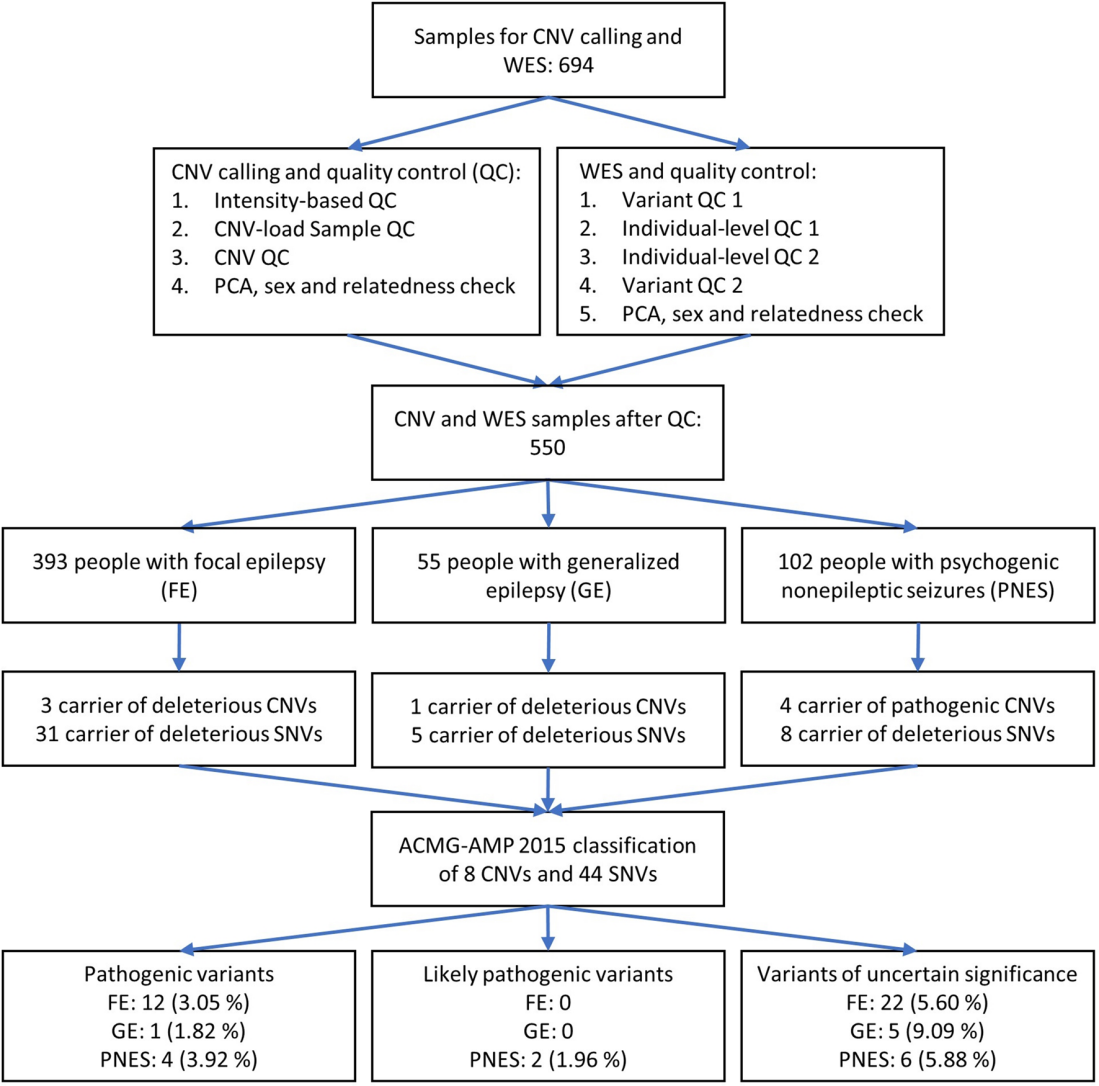
Study design and execution. SNV: single nucleotide variant, CNV: copy number variant, WES: whole-exome sequencing, QC: quality control, PCA: principal component analysis, FE: focal epilepsy, GE: generalized epilepsy, PNES: psychogenic nonepileptic seizures.

To characterize genetically the heterogeneous phenotypic spectrum of individuals referred to a tertiary care center—we genotyped and sequenced 550 individuals with PNES or epilepsy, from the Cleveland Clinic Epilepsy Center, while excluding individuals with both PNES and epilepsy. We then selected all copy-number (CNVs) and single nucleotide variants (SNVs) with strong computational support for a deleterious effect on the involved gene/s and classified them according to the American College of Medical Genetics and Genomics and the Association for Molecular Pathology (ACMG-AMP) 2015 guidelines^13^. The guidelines require an established gene to phenotype association as one criterion for pathogenicity prediction. Because the genetic basis of PNES is unknown and no established PNES genes exist, we applied the ACMG-AMP guidelines for research purposes only and considered genes associated with neurological or psychiatric disorders as candidate genes for PNES. Our overall study design is presented in Fig. 1.

## Results

### Cohort and analysis overview

We generated genotyping as well as exome data for 694 individuals ascertained through the Epilepsy Biorepository of the Cleveland Clinic Epilepsy Center. The data was used to perform genome-wide CNV and whole-exome SNV analyses. After data quality control, 550 individuals with epilepsy or PNES were included in the downstream analyses (Table 1). The most prevalent diagnosis was focal epilepsy (FE, N = 393, 71.5%) followed by psychogenic nonepileptic seizures without epilepsy (PNES, N = 102, 18.5%), and generalized epilepsy (GE, N = 55, 10%).

**Table 1:** Demographic and clinical features of the 550 tertiary care epilepsy center patients.

	PNES	FE	GE	F/χ^2^	*P*
	N = 102	N = 393	N = 55		
	M (SD)	M (SD)	M (SD)		
Age	42.5 (14.3)	39.7 (14.8)	38.2 (15.0)	2.03	0.133
Education	13.7 (2.4)	13.9 (2.5)	14.1 (2.2)	0.51	0.602

	PNES	FE	GE	F/χ^2^	*P*
	N = 102	N = 393	N = 55		
	N (%)	N (%)	N (%)		
Sex (female)	76 (74.5%)	183 (46.6%)	31 (56.4%)	25.7	< 0.001
**Comorbidities and family history**					
Depression	64 (62.7%)	164 (41.7%)	30 (54.5%)	15.8	< 0.001
Anxiety	54 (52.9%)	106 (27.0%)	25 (45.5%)	28.3	< 0.001
Bipolar disorder	11 (10.8%)	1 (1.8%)	1 (1.8%)	20.2	< 0.001
PTSD	14 (13.7)	5 (1.3%)	1 (1.8%)	36.4	< 0.001
Chronic pain	18 (17.6%)	23 (5.9%)	4 (7.3%)	15.1	0.001
Family history of seizures	32 (31.4%)	78 (19.8%)	20 (36.4%)	11.4	0.003

PNES: psychogenic nonepileptic seizures, FE: focal epilepsy, GE: generalized epilepsy, M: sample mean, SD: standard deviation, F/χ^2^: test statistic, *P*: *P*-value, N: number of individuals, PTSD: post-traumatic stress disorder.

Individuals in this study were 40 years old on average (standard deviation, SD = 15) and completed approximately 14 years of education (SD = 2). Fifty-three percent of the cohort was female and all of genetically defined European ancestry. The three study groups were well matched in terms of age and education. There were more females in the PNES group (75%) than in the epilepsy groups (FE: 47%, GE: 56%). The PNES group showed the highest proportion of individuals with comorbid psychiatric disorders or a history of chronic pain compared to the two epilepsy groups (Table 1). Individuals with FE were less likely to have a family history of epilepsy, defined as seizures in a first- or second-degree relative than those with GE (χ^2^ = 7.70, *P* = 0.006) or PNES (χ^2^ = 6.22, *P* = 0.013). However, the family history of seizures was similar between individuals with GE and PNES (χ^2^ = 0.402, *P* = 0.526). The percentage of individuals with comorbidities and a family history of seizures was similar between females and males with PNES (Supplementary Table 4).

### Individuals with PNES carry pathogenic and likely pathogenic variants similar to individuals with epilepsy

**Figure 2:**
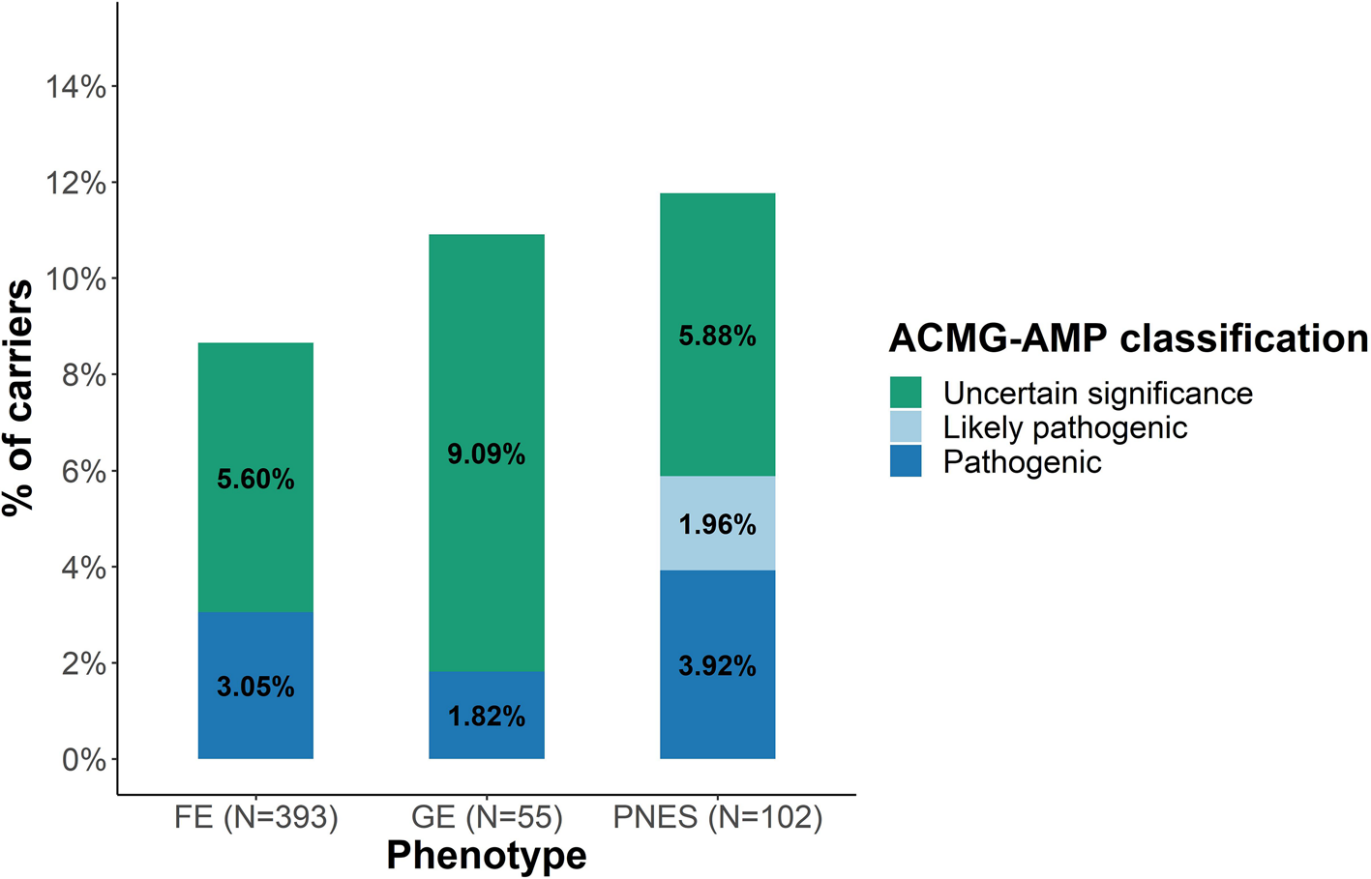
Burden of pathogenic and likely pathogenic SNVs and CNVs in individuals with FE, GE, or PNES. Each stacked bar plot represents the total percentage of carriers of (i) pathogenic variants, highlighted in blue; (ii) likely pathogenic variants, highlighted in light blue; and (iii) variants of uncertain significance, highlighted in green. The classification of the variants in the individuals with PNES was performed for research purposes only. FE: focal epilepsy, GE: generalized epilepsy, PNES: psychogenic nonepileptic seizures, N: number of individuals with each phenotype.

Rare genetic variants are well-established risk factors associated with epilepsy^14^. We first examined if individuals with PNES carry deleterious genetic variants, as classified by state-of-the-art in silico prediction methods. The results were compared to the control group of 448 individuals with epilepsy from the same epilepsy clinic. Identified potential deleterious insertion/deletion polymorphisms (indels, N = 56) did not survive our strict criteria for the visual inspection of the reads supporting each variant and were excluded from subsequent analyses. After data quality control and stringent variant filtering, we identified deleterious variants in 8.65% of all individuals with FE (N = 34, Supplementary Table 1), 10.91% of all individuals with GE (N = 6, Supplementary Table 2), and in 11.76% of all individuals with PNES (N = 12, Table 2) (Fig. 2). The proportions of individuals carrying only a specific variant type (single nucleotide or copy number variants) are displayed in Supplementary Figures 1 and 2. The deleterious variant burden across the three disorder types was not significantly different (PNES vs. FE vs. GE, two-tailed 3 × 2 χ^2^, *P* = 0.59; PNES vs. epilepsy, two-tailed 2 × 2 χ^2^, *P* = 0.38).

**Table 2:** Pathogenic, likely pathogenic, and variants of uncertain significance in individuals with PNES.

ID	Variant type	Gene/s	Associated neurological/psychiatric disorder	ACMG-AMP classification	CytoBand	Chr	Start GRCh37	Stop GRCh37	Consequence/nucleotide change/affected exon	Mis_z	pLI
PNES1	1.58 Mb deletion	7 genes	Epilepsy	Pathogenic	10q23.1-q23.2	10	87,136,787	88,718,934	CN loss	–	–
PNES2	218 Kb deletion	9 genes	Epilepsy	Pathogenic	distal 16p11.2	16	28,826,049	29,044,745	CN loss	–	–
PNES3	229 Kb deletion	*PAFAH1B1, METTL16*	Epilepsy	Pathogenic	17p13.3	17	2,341,350	2,570,479	CN loss	–	–
PNES4	Nonsynonymous SNV	NSD1	Neurological/psychiatric	Likely pathogenic	5q35.3	5	176,720,953	176,720,953	p.Lys1926Arg (exon 23 out of 23)	3.70	1.00
PNES5	4.91 Mb Deletion	39 genes	Neurological/psychiatric	Pathogenic	10q11.22-q11.23	10	46,943,377	51,856,375	CN loss	–	–
PNES6	Nonsynonymous SNV	*GABRA5*	Neurological/psychiatric	Likely pathogenic	15q12	15	27,188,485	27,188,485	p.Ala334Gly (exon 10 out of 11)	3.31	0.88
PNES7	Stopgain SNV	*LHX9*	NA	Uncertain significance	1q31.3	1	197,896,819	197,896,819	p.Gln269Ter (exon 4 out of 5)^a^	1.13	0.98
PNES8	Nonsynonymous SNV	*MAPKAPK2*	NA	Uncertain significance	1q32.1	1	206,904,045	206,904,045	p.Leu235Pro (exon 6 out of 10)	3.10	1.00
PNES9	Splicing SNV	*CAMKV*	NA	Uncertain significance	3p21.31	3	49,899,726	49,899,726	c.95+1G>A (exon 2 out of 11)	3.39	1.00
PNES10	Stopgain SNV	*GAPVD1*	NA	Uncertain significance	9q33.3	9	128,118,066	128,118,066	p.Arg1319Ter (exon 25 out of 28) ^b^	2.75	1.00
PNES11	Nonsynonymous SNV	*PRPF8*	NA	Uncertain significance	17p13.3	17	1,579,337	1,579,337	p.Arg855Pro (exon 18 out of 43)	8.55	1.00
PNES12	Splicing SNV	*MYH9*	NA	Uncertain significance	22q12.3	22	36,717,867	36,717,867	c.706-1G>C (exon 7 out of 41)	3.67	1.00

Chr: chromosome, GRCh37: Genome Reference Consortium Human Build 37, Mis_z: Z-score for missense variant intolerance of a gene, pLI: probability for the loss-of-function variant intolerance of a gene, Mb: mega base pairs, Kb: kilo base pairs, CN: copy number.

^a^Exon numbers based on transcript NM_020204.

^b^Exon numbers based on transcript NM_001282680.

Next, we classified all deleterious variants according to the ACMG-AMP 2015 guidelines to determine if individuals with PNES carry ACMG-AMP-classified pathogenic (P) or likely pathogenic (LP) genetic variants in genes associated with neurological disorders, and/or psychiatric disorders, including epilepsy. We found P/LP variants in 3.05% of all individuals with FE (N = 12, Supplementary Table 1), 1.82% of all individuals with GE (N = 1, Supplementary Table 2), and in 5.88% of all individuals with PNES (N = 6, Table 2) (Fig. 2). The P/LP variant burden across the three disorder types was not significantly different (PNES vs. FE vs. GE, two-tailed 3 × 2 χ^2^, *P* = 0.30; PNES vs. epilepsy, two-tailed 2 × 2 χ^2^, *P* = 0.14). We also identified ACMG-AMG variants of uncertain significance (VUS) in 5.60% of all individuals with FE (N = 22, Supplementary Table 1), 9.09% of all individuals with GE (N = 5, Supplementary Table 2), and in 5.88% of all individuals with PNES (N = 6, Table 2) (Fig. 2). Out of all six P/LP variant carriers with PNES, one (16.7%) reported a family history of seizures, and two (33.3%) a family history of psychiatric disorders (anxiety, mood disorders, and post-traumatic stress disorder).

### Detailed evaluation of the deleterious variants found in individuals with PNES

We are unaware of any studies to date that have conducted genetic testing in individuals with PNES. Subsequently, there are no genes or variants that have been associated with PNES. Variant pathogenicity classification requires an established gene-phenotype association^13^. For research purposes, we classified variants in our sample of individuals with PNES as if the variants would have been identified in individuals with neurologic or psychiatric disorders. As a consequence, variants in genes associated with neuropsychiatric disorders could qualify for pathogenicity if additional variant level criteria were fulfilled.

The phenotypic characteristics of all deleterious variant carriers with PNES are listed in Table 3 and Supplementary Tables 5 and 6. Six individuals with PNES were affected by P/LP variants (three males and three females). These variants included four CNVs and two missense variants (Table 2). Three out of the six P/LP variants were found to be known epilepsy-associated CNVs (deletions at 10q23.1-q23.2, distal 16p11.2, and 17p13.3). Deletions at 10q23.1-q23.2 are associated with complex genetic neurodevelopmental syndromes^15^. Our patient with PNES and the 10q23.1-q23.2 deletion had normal neurodevelopment, an onset of PNES at 33 years of age, a history of migraine, chronic pain and mood disorder, and no history of febrile seizures or family history of seizures.

**Table 3:** Phenotypic characteristics of individuals with PNES and deleterious genetic variants.

ID	Sex	Identified variant (ACMG-AMP classification)	Age	Age at PNES onset	Cognition/neuro exam	MRI	Febrile seizures	Medical comorbidities	Psychiatric comorbidities	FH of seizures	FH of psychiatric disease
PNES1	Male	Pathogenic	33	33	Normal/normal	Normal	No	Migraine, chronic pain	Mood disorder	No	No
PNES2	Male	Pathogenic	59	55	Normal/normal	Normal	No	HTN, DM, HPL, COPD, OSA, CAD/CHF, CKD	None	No	No
PNES3	Female	Pathogenic	33	30	Normal/normal	Unknown	No	None	Anxiety, PTSD	No	Yes—PTSD, anxiety
PNES4	Male	Likely pathogenic	58	12	Normal/normal	Normal	No	HTN, OSA	Mood disorder	No	No
PNES5	Female	Pathogenic	50	50	Normal/normal	Normal	No	Hypothyroidism	Mood disorder, anxiety	Yes—aunt	Yes—mood disorder
PNES6	Female	Likely pathogenic	45	40	Normal/normal	Normal	No	HTN, HPL, CAD, CVD	Mood disorder, anxiety, PTSD	No	No
PNES7	Female	Uncertain significance	39	34	Normal/normal	Normal	No	Migraine, HTN, DM, OSA, PCOS, optic neuritis	Mood disorder, anxiety	No	Yes—mood disorder, OCD
PNES8	Female	Uncertain significance	50	50	Normal/normal	Normal	No	Migraine, HPL	Anxiety	No	No
PNES9	Female	Uncertain significance	48	28	Normal/normal	Mild chronic microvascular disease	No	DM, OSA, IBS, CVD	Mood disorder, anxiety	No	No
PNES10	Female	Uncertain significance	43	7	Normal/normal	Mild chronic microvascular disease	No	Migraine, DM, CKD, OSA, hypothyroidism	Mood disorder, anxiety	Yes—MGM	No
PNES11	Female	Uncertain significance	26	15	Normal/normal	Normal	No	Migraine, chronic pain	Mood disorder, anxiety, PTSD	Yes—MGF	Yes—mood disorder, anxiety
PNES12	Male	Uncertain significance	53	51	Normal/normal	Normal	No	None	Mood disorder	Yes—mother	No

HTN: hypertension, DM: diabetes mellitus, HPL: hyperlipidemia, COPD: chronic obstructive pulmonary disease, CAD: coronary artery disease, CHF: congestive heart failure, CKD: chronic kidney disease, OSA: obstructive sleep apnea, PTSD: post-traumatic stress disorder, Mood Disorder: depression or bipolar disorder, CVD: cerebrovascular disease, PGF: paternal grandfather, PCOS: polycystic ovarian syndrome, OCD: obsessive–compulsive disorder, MGF: maternal grandfather, MGM: maternal grandmother, IBS: irritable bowel syndrome.

Deletions and duplications at distal 16p11.2 locus are associated with epilepsy, autism spectrum disorder, schizophrenia, lower IQ, and subcortical brain abnormalities^16^. Our patient with PNES and the distal 16p11.2 deletion had normal neurodevelopment, an onset of PNES at 55 years of age, and no history of febrile seizures or a family history of seizures. The third epilepsy-associated variant in PNES was a *PAFAH1B1* gene disrupting 17p13.3 deletion. *PAFAH1B1* is a well-established gene for lissencephaly with seizures as the core symptom of this disorder^17^. The 17p13.3 deletion also disrupts the gene *METTL16*. However, *METTL16* is not associated with any disease and is unlikely to play a role in PNES causation, because *METTL16* is known to tolerate missense (missense Z-score = 2.35) and loss-of-function variants (pLI = 0.61)^18^. Our patient with PNES and deletion of *PAFAH1B1* had normal neurodevelopment, an onset of PNES at 30 years of age, a history of anxiety and post-traumatic stress disorder (PTSD), and no history of febrile seizures or family history of seizures.

The next three P/LP variants (one P, two LP) were identified in two genes and at a chromosomal position, each implicated in neurological or psychiatric disorders with or without seizures (*NSD1*, 10q11.22-q11.23 deletion, and *GABRA5*; Table 2). Heterozygous mutations in the *NSD1* gene are associated with Sotos syndrome (autosomal dominant)^19^. Sotos syndrome is characterized by distinctive facial features, intellectual disability (ID), and overgrowth or macrocephaly, and seizures are reported in 9–50% of cases^20^. Our patient with PNES and an *NSD1* mutation had normal neurodevelopment, an onset of PNES at 12 years of age, a history of mood disorder, and no history of febrile seizures or family history of seizures. Deletions and duplications at 10q11.22-q11.23 are associated with developmental delay or ID^21^. Our patient with PNES and the 10q11.22-q11.23 deletion had normal neurodevelopment, an onset of PNES at 50 years of age, a history of mood disorder and anxiety, no history of febrile seizures, and a suggestive family history of seizures (affected aunt). Finally, *GABRA5* is a candidate gene for epilepsy and developmental delay^22^, which has not yet been statistically associated with epilepsy through an exome-wide cohort screen. Our patient with PNES and a *GABRA5* mutation had normal neurodevelopment, an onset of PNES at 40 years of age, a history of mood disorder, anxiety, and PTSD, and no history of febrile seizures or family history of seizures.

Out of the six identified VUS, four were identified in genes with a potential role in brain function (*LHX9, MAPKAPK2, CAMKV,* and *MYH9*). Expression of *LHX9* was shown to be repressed by kainic acid-induced seizures^23^, while *MAPKAPK2* expression was induced^24^. *CAMKV* encodes for a synaptic protein crucial for dendritic spine maintenance^25^. Heterozygous mutations in *MYH9* are associated with a spectrum of autosomal dominant thrombocytopenias^26^. The most devastating consequence of thrombocytopenia is intracranial hemorrhage, and one case with recurrent seizures related to intracranial hemorrhage was recently described in the literature^27^.

## Discussion

We generated whole-exome sequencing and whole-genome genotyping data to identify rare, P/LP variants in a cohort of 550 individuals with PNES or epilepsy (focal or generalized). This study represents the first genetic investigation of PNES. We used the ACMG-AMP 2015 guidelines^13^ to classify variants from the perspective of Mendelian forms of neurological or psychiatric disorders, including epilepsy. We show that P/LP variants in genes implicated in a broad range of neurological and psychiatric disorders are found in individuals with PNES evaluated in a tertiary care epilepsy center. Interestingly, we did not observe a significant difference in the burden of P/LP variants, when comparing individuals with PNES without coexistent epilepsy to individuals with epilepsy. The observed variant burden is not surprising for the epilepsies, and elevated burdens of CNVs or SNVs have been observed in several large-scale studies that compared individuals with epilepsy against population controls^11,14,28,29^. In contrast, genetic factors have not been previously identified for PNES.

We executed a very stringent state-of-the-art variant filtering strategy, optimized for high specificity to identify deleterious SNVs. Following evidence from large-scale studies in epilepsy and other neurological disorders showing that most of the causal variants are ultra-rare in the general population^14,30^, we only selected and classified unique SNVs not seen in > 200 k population controls. We only considered CNVs if the locus had previously been associated with neurological or psychiatric disorders. The goal of this strategy was to prioritize genetic variants that have a high likelihood to be true and pathogenic for a robust comparison of the PNES and epilepsy groups. Future studies will have to balance sensitivity vs. specificity to prioritize variant discovery.

Among the 12 deleterious variants identified in individuals with PNES without coexistent epilepsy, 50% (6/12) were classified according to the ACMG-AMP 2015 guidelines as pathogenic or likely pathogenic. The ACMG-AMP 2015 guidelines require an established gene to phenotype association as one criterion for pathogenicity prediction^13^. In a clinical setting, the same variants would not be classified as pathogenic since no single established PNES gene exists. Nevertheless, the detection of pathogenic variants that are likely to cause Mendelian forms of neurological or psychiatric disorders in individuals with PNES is in line with emerging evidence that neurological or psychiatric disorders share a broad range of pleiotropic acting genetic variation^8,11,31^. Our results are also in line with the observation that up to 48% of all individuals with PNES report a family history of epilepsy and 22% a family history of psychiatric disorders^6^. PNES could represent one of several clinically defined phenotypes associated with pleiotropic acting genetic variants, affecting genes essential for brain development and function. Surprisingly, none of the P/PL variant carriers with PNES had an abnormal bedside cognition/neurological examination or any major structural abnormality on MRI. However, formal neuropsychological testing was not performed; therefore, it is possible that individuals had more subtle forms of cognitive impairment. It is also possible that our results represent phenotype expansions for some of the genes affected by P/PL variants. Phenotype expansions are common as shown for *SLC6A1*, a known gene for autism spectrum disorders, epilepsy, and ID^32^, which was recently associated with schizophrenia without ID or other neurodevelopmental disorder^33^. Finally, despite an ACMG-AMP 2015 guidelines classification as VUS, we cannot exclude the involvement of the six identified VUS in PNES causation, as all variants identified in this study were predicted in silico as highly pathogenic, never seen in the general population, and affected highly variant intolerant genes. Future case/control studies are needed to identify genes that have not been implicated in other disorders as associated with PNES.

The four identified deletions in individuals with PNES in this study have been reported in the literature in epilepsy and other neurological disorders, with high phenotypic variability and incomplete penetrance^15,16,21,34^. This high degree of pleiotropy has been observed for the vast majority of all deletions that have been associated with epilepsy or other neurological disorders^35^. Most likely, these deletions impair neurodevelopmental processes in a rather nonspecific manner and contribute to the genetic variance of a broad spectrum of neurological disorders. The specific disease phenotype is likely further specified by the interplay with genetic background effects and environmental influences following an oligo-/polygenic inheritance model with substantial genetic heterogeneity. Environmental factors which predispose to PNES are very well established^1^. Genetic vulnerability for a neurological or psychiatric condition could predispose to PNES when combined with environmental stressors. Individual differences in the genetic vulnerability to specific types of trauma and other environmentally relevant variables have been demonstrated, for example, for PTSD^36,37^. In our study, the P/LP variant carriers showed a similar rate of presence/absence of a history of trauma or abuse as individuals with PNES and no identified variants (P/LP carriers: 3/3 vs. no variants identified: 30/25). However, more research is needed to support this hypothesis and to explain our observation that individuals with PNES alone can carry pathogenic variants that affect genes linked to clinically severe phenotypes.

In conclusion, in this report, we provide the first evidence that genetic factors may play a role in the etiology of PNES. Future large-scale projects that employ comprehensive genetic testing, including polygenic risk scores, are needed for PNES and related genetically understudied disorders such as PTSD, and other conversion disorders. Potentially, such work could provide clues to the etiology and pathophysiology of the disorder, enable the identification of disease biomarkers, and set new directions for the development of new therapies for PNES.

## Material and methods

### Study participants

Data for this study were obtained from a Cleveland Clinic Institutional Review Board-approved epilepsy biorepository. All methods were performed in accordance with relevant guidelines and regulations regarding research involving human subjects. All participants provided written, informed consent, and a blood or saliva sample for use in medical/genetic research. Participants were selected for study inclusion if they met the following criteria: (1) age 18 years or older, (2) video-EEG confirmed diagnosis of focal epilepsy (FE), generalized epilepsy (GE), or psychogenic nonepileptic seizures (PNES) without comorbid epileptic seizures, and (3) had blood or saliva DNA available for whole-exome sequencing (N = 694). Our study cohort is detailed in Fig. 1.

### Phenotyping procedures

Initial medical record review was conducted for all potential study participants by an epilepsy biorepository research coordinator trained in clinical epilepsy phenotyping (L.F.) and supervised by clinical epileptologists (J.F.B., L.J.). Diagnoses of epilepsy or PNES were established after review of the history and physical examination, scalp EEG video evaluation report, progress notes, social work, psychology and/or psychiatry report, and discharge summary as well as other documentation pertinent to the medical history and clinical diagnoses. Psychiatric diagnoses were established through review of the medical records and patient self-reports during the video-EEG admission. Individuals were classified as having FE, GE, or PNES based on video-EEG and concordant history. All individuals classified as PNES on initial review were re-reviewed by a clinical epileptologist (J.F.B.) to confirm the diagnosis. The diagnosis of PNES required video-EEG recording of a semiology typical for PNES, and an absence of epileptiform abnormalities on either interictal or ictal EEG^38^. Accordingly, all individuals with PNES included in this study had video-EEG diagnosed PNES without comorbid epilepsy. Individuals with episodes explained by another diagnosis such as physiologic nonepileptic events (e.g., syncope, migraine, sleep disorder) were excluded from the study as were those with comorbid PNES and epileptic seizures and those with episodes consisting of purely subjective symptoms without objective signs (to prevent the inclusion of individuals with epileptic auras, which often do not have EEG abnormalities).

### Copy number calling

A total of 688,032 single nucleotide polymorphisms (SNPs) were genotyped for all samples of this study using the Global Screening Array with Multi-disease drop-in (GSA-MD v1.0) (Illumina, San Diego, CA, USA). The SNP-data was used to detect CNVs in our dataset, using PennCNV’s copy number variant (CNV) calling algorithm^39^ with GC-wave adjustment. We generated a custom population B-allele frequency (BAF) file before calling CNVs. Adjacent CNVs were merged if the number of intervening markers between them was less than 20% of the total number of the whole segment encompassing both CNVs. CNV calling was followed by extensive quality control (QC) for both samples and CNVs, respectively. Samples with signal intensity log R Ratio (LRR) standard deviation < 0.23, variability of the average LRR values in sliding windows (waviness factor, WF) < 0.02, departure of the BAF from the expected values for two copies (BAF drift) < 0.003, total numbers of CNVs < 80, and European ancestry were included in the analysis. CNV calls were removed from the dataset if they spanned less than 20 markers, were less than 20 Kb in length, had a marker density (amount of markers/length of CNV) < 0.0001, overlapped by > 50% of their total length with regions known to generate artifacts^40^, or had a frequency > 1% in the study sample. CNVs that were spanning more than 20 markers over ≥ 1 Mb were included in the analysis, even if the marker density was < 0.0001. All CNVs of interest were examined visually by plotting the signal intensities using PennCNV^39^ (Supplementary Figures 3–9).

### Whole-exome sequencing

Whole-exome sequencing (WES) of all samples in this study was performed using Nextera Rapid Capture Exomes enrichment and paired-end reads (151 bp) Illumina sequencing on the Illumina HiSeq 4,000. Duplicate read removal, format conversion, and indexing of the reads, aligned to the GRCh37 human genome reference (RefSeq: GCF_000001405.13), were performed using Picard (https://broadinstitute.github.io/picard). All samples were jointly called using the Genome Analysis Toolkit (GATK) best practice pipeline^41^.

### WES quality control

Quality control (QC) was performed in two iterations of a sample- and variant-level quality filtering. Sequencing and alignment quality metrics were computed using Picard tools (https://broadinstitute.github.io/picard/), leading to the exclusion of samples with freemix contamination estimates > 0.02 and excess chimeric reads > 1%. Low quality variants were filtered out based on the following criteria: (i) phred quality score, QUAL < 20; (ii) GATK truth sensitivity tranche > 99.5% for single nucleotide variants (SNVs) and > 95% for indels; (iii) > 2 alleles; (iv) inbreeding coefficient < − 0.2; (v) sample read depth (DP) for SNVs < 20 and for indels < 30; (vi) genotype quality (GQ) < 99; (vii) allelic balance of heterozygous SNV < 0.25, homozygous SNV < 0.9, heterozygous indels < 0.30, and homozygous indels < 0.95. At the individual level, we removed related samples (KING^42^ kinship coefficient > 0.0442), samples not clustering with the 1,000 Genomes Project European-ancestry samples (GCTA^43^ principal component analysis), and samples with ambiguous sex or mismatch with the reported gender. We also filtered out samples that exceeded three standard deviations (SD) from the mean of the entire study cohort on any of the following WES metrics: (i) low mean DP; (ii) low singleton count; (iii) low SNV count; (iv) low or high singleton/SNV ratio; (v) low transition/transversion ratio; (vi) low or high heterozygous/homozygous variant ratio; and (vii) low or high insertion/deletion ratio. Finally, we applied the following exclusion thresholds for variants in the samples that survived previous QC filtering: (i) genotype call rate < 0.95; (ii) minor allele frequency > 0.1; (iii) deviation from the Hardy–Weinberg equilibrium with *P* < 1 × 10^–20^. The supporting aligned reads of all variants that survived filtering for deleteriousness, detailed in the following paragraph, were visually inspected using the IGV browser^44^.

### Variant deleteriousness assessment

We applied for the identified CNVs and SNVs two different strategies to assess the likelihood of a deleterious effect on disease-relevant loci or genes. CNVs: We only considered CNVs as deleterious if the locus had previously been associated with neurological or psychiatric disorders. SNVs: We used ANNOVAR^45^ with custom databases to perform a state-of-the-art variant annotation for subsequent deleteriousness assessment. We applied two different filters, based on: i) variant type and frequency and (ii) predicted variant deleteriousness. The frequency-based filter was based on the following criteria: (i) variant not in a genomic duplication (> 1,000 bases of non-repeat masked sequence); (ii) not present in multiple variant databases, totaling > 200 k population controls (Supplementary Table 3). From the remaining variants, we selected only variants with a high confidence prediction to be deleterious using following criteria: (i) loss-of-function (LoF) variants ranked in the top 1% most deleterious variants in the human genome (scaled CADD ≥ 20)^46^ found in known epilepsy genes or highly LoF-intolerant genes^18^ (probability, pLI ≥ 0.95); (ii) missense variants ranked in the top 1% most deleterious variants in the human genome (scaled CADD ≥ 20)^46^ found in missense-constrained regions (MPC ≥ 2 and MTR centile < 15%)^47,48^, of epilepsy genes or missense intolerant genes (missense Z-score > 3.09, corresponding to *P* < 10^–3^)^18^.

### ACMG-AMP 2015 classification of the deleterious CNVs and SNVs

The pathogenicity of all deleterious SNVs was assessed according to the ACMG-AMP guidelines^13^ using InterVar^49^. We adjusted the InterVar interpretations were needed for: (i) variant type in genes with a known mode of inheritance; (ii) location of the variant in protein; (iii) absence of variant in population controls; (iv) known gene function and association with disease. Deleterious SNVs expected to cause disorders that are not neurological or psychiatric (i.e., incidental findings), were considered as variants of uncertain significance. The pathogenicity of all deleterious CNVs was assessed directly, using the ACMG-AMP criterions PVS1, PS1, and PS4^13^, when supported by definite evidence.

## Supplementary information

Supplementary Information 1.

## Data Availability

Data are available from the corresponding authors on reasonable request.
