# Supplementary material for: Neurological disorder-associated genetic variants in individuals with psychogenic nonepileptic seizures
Source: Sci Rep. 2020 Sep 16;10:15205. doi: 10.1038/s41598-020-72101-8 (PMC7495430; doi:10.1038/s41598-020-72101-8)
Supplement: Supplementary file 1 — Supplementary Information 1. [file 41598_2020_72101_MOESM1_ESM.docx]

**Supplementary Information**

**Neurological disorder-associated genetic variants in individuals with psychogenic nonepileptic seizures**

Costin Leu,^1,2,3*^ Jocelyn F. Bautista,^4,5^ Monica Sudarsanam,^1^ Lisa-Marie Niestroj,^6^ Arthur Stefanski,^1^ Lisa Ferguson,^4,5,7^ Mark J. Daly,^2,8,9^ Lara Jehi,^4,5^ Imad M. Najm,^4,5^ Robyn M. Busch,^4,5,7^ Dennis Lal^1,2,4,6*^

1. Genomic Medicine Institute, Lerner Research Institute, Cleveland Clinic, Cleveland, OH 44195, US
2. Stanley Center for Psychiatric Research, Broad Institute of Harvard and M.I.T, Cambridge, MA 02142, US
3. Department of Clinical and Experimental Epilepsy, Institute of Neurology, University College London, Queen Square, London WC1N 3BG, UK
4. Epilepsy Center, Neurological Institute, Cleveland Clinic, Cleveland, OH 44195, US
5. Department of Neurology, Neurological Institute, Cleveland Clinic, Cleveland, OH 44195, US
6. Cologne Center for Genomics (CCG), University of Cologne, 50931 Cologne, DE
7. Department of Psychiatry & Psychology, Neurological Institute, Cleveland Clinic, Cleveland, OH 44195, US
8. Institute of Molecular Medicine Finland (FIMM), Helsinki Institute of Life Science (HiLIFE), University of Helsinki, Helsinki, FI
9. Analytic and Translational Genetics Unit, Massachusetts General Hospital, Boston, MA 02114, US

* **Corresponding authors:**

Costin Leu, PhD

Genomic Medicine Institute

Lerner Research Institute

Cleveland Clinic

Cleveland, OH 44195, US

Email: leuc@ccf.org

Dennis Lal, PhD

Genomic Medicine Institute

Lerner Research Institute

Cleveland Clinic

Cleveland, OH 44195, US

Email: lald@ccf.org

1. **Supplementary Tables**
   1. Supplementary Table 1: Pathogenic, likely pathogenic, and variants of uncertain significance in individuals with focal epilepsy
   2. Supplementary Table 2: Pathogenic, likely pathogenic, and variants of uncertain significance in individuals with generalized epilepsy
   3. Supplementary Table 3: Public variant databases
   4. Supplementary Table 4: Percentage of females and males with PNES and comorbid disorders or a family history of seizures
   5. Supplementary Table 5: History of trauma or abuse and risk factors for epilepsy in the P/LP variant and VUS carriers with PNES
   6. Supplementary Table 6: Summary statistics of the history of trauma or abuse and risk factors for epilepsy in all individuals with PNES
2. **Supplementary Figures**
   1. Supplementary Figure 1: Burden of copy number variations (CNVs)
   2. Supplementary Figure 2: Burden of single nucleotide variants (SNVs)
   3. Supplementary Figure 3: 10q23.1-q23.2 deletion identified in an individual with PNES (ID: PNES1)
   4. Supplementary Figure 4: Distal 16p11.2 deletion identified in an individual with PNES (ID: PNES2)
   5. Supplementary Figure 5: 17p13.3 deletion identified in an individual with PNES (ID: PNES3)
   6. Supplementary Figure 6: 16p12.2 deletion identified in an individual with FE (ID: FE4)
   7. Supplementary Figure 7: 16p11.2 deletion identified in an individual with FE (ID: FE5)
   8. Supplementary Figure 8: 17q11.2 deletion identified in an individual with FE (ID: FE6)
   9. Supplementary Figure 9: 16p13.11-p12.3 deletion identified in an individual with GE (ID: GE1)
3. **References**

**1. Supplementary Tables**

**Supplementary Table 1: Pathogenic, likely pathogenic, and variants of uncertain significance in individuals with focal epilepsy**

| **ID** | **Variant type** | **Gene/s** | **Associated neurological / psychiatric disorder** | **ACMG-AMP classification** | **CytoBand** | **Chr** | **Start_GRCh37** | **Stop_GRCh37** | **Consequence / nucleotide change** | **Mis_z** | **pLI** |
| --- | --- | --- | --- | --- | --- | --- | --- | --- | --- | --- | --- |
| FE1 | stopgain SNV | SZT2 | epilepsy | Pathogenic | 1p34.2 | 1 | 43898269 | 43898269 | p.Gln1785Ter | 2.61 | 0.00 |
| FE2 | stopgain SNV | KCNH1 | epilepsy | Pathogenic | 1q32.2 | 1 | 211192515 | 211192515 | p.Cys214Ter | 3.94 | 0.62 |
| FE3 | stopgain SNV | ASAH1 | epilepsy | Uncertain significance | 8p22 | 8 | 17942254 | 17942254 | p.Tyr19Ter | -2.14 | 0.00 |
| FE4 | 458Kb Deletion | 7 genes | epilepsy | Pathogenic | 16p12.2 | 16 | 21956457 | 22414463 | CN loss | - | - |
| FE5 | 572Kb Deletion | 27 genes | epilepsy | Pathogenic | 16p11.2 | 16 | 29627349 | 30199713 | CN loss | - | - |
| FE6 | 674Kb Deletion | 11 genes | epilepsy | Pathogenic | 17q11.2 | 17 | 29111368 | 29785784 | CN loss | - | - |
| FE7 | stopgain SNV | MTOR | neurological / psychiatric | Pathogenic | 1p36.22 | 1 | 11188962 | 11188962 | p.Glu1921Ter | 7.38 | 1.00 |
| FE8 | splicing SNV | HECW2 | neurological / psychiatric | Pathogenic | 2q32.3 | 2 | 197085667 | 197085667 | c.4147-2A>G | 3.43 | 1.00 |
| FE9 | stopgain SNV | MED12L | neurological / psychiatric | Pathogenic | 3q25.1 | 3 | 151085982 | 151085982 | p.Arg1130Ter | 3.26 | 1.00 |
| FE10 | splicing SNV | CLOCK | neurological / psychiatric | Uncertain significance | 4q12 | 4 | 56315037 | 56315037 | c.1450-2A>C | 1.95 | 1.00 |
| FE11 | stopgain SNV | NDST1 | neurological / psychiatric | Pathogenic | 5q33.1 | 5 | 149907393 | 149907393 | p.Gln181Ter | 2.97 | 1.00 |
| FE12 | splicing SNV | KCNQ5 | neurological / psychiatric | Pathogenic | 6q13 | 6 | 73751787 | 73751787 | c.616+2T>G | 3.45 | 0.98 |
| FE13 | stopgain SNV | LRRC4 | neurological / psychiatric | Pathogenic | 7q32.1 | 7 | 127670199 | 127670199 | p.Tyr165Ter | 1.89 | 1.00 |
| FE14 | nonsynonymous SNV | CACNA1I | neurological / psychiatric | Uncertain significance | 22q13.1 | 22 | 40042676 | 40042676 | p.Arg418Cys | 5.32 | 1.00 |
| FE15 | stopgain SNV | FLNA | neurological / psychiatric | Pathogenic | Xq28 | X | 153579318 | 153579318 | p.Ser2372Ter | 3.84 | 1.00 |
| FE16 | stopgain SNV | EYA3 | NA | Uncertain significance | 1p35.3 | 1 | 28362109 | 28362109 | p.Gln103Ter | 2.02 | 1.00 |
| FE17 | stopgain SNV | SPHKAP | NA | Uncertain significance | 2q36.3 | 2 | 228881885 | 228881885 | p.Arg1229Ter | -0.63 | 0.96 |
| FE18 | stopgain SNV | NFKBIZ | NA | Uncertain significance | 3q12.3 | 3 | 101572617 | 101572617 | p.Ser316Ter | 1.44 | 1.00 |
| FE19 | stopgain SNV | FBN2 | NA | Uncertain significance | 5q23.3 | 5 | 127614418 | 127614418 | p.Trp2418Ter | 1.99 | 1.00 |
| FE20 | splicing SNV | TCOF1 | NA | Uncertain significance | 5q32 | 5 | 149756184 | 149756184 | c.2340+1G>T | 0.33 | 0.99 |
| FE21 | nonsynonymous SNV | GRIA1 | NA | Uncertain significance | 5q33.2 | 5 | 153144139 | 153144139 | p.Thr562Ala | 3.68 | 1.00 |
| FE22 | nonsynonymous SNV | ADCY1 | NA | Uncertain significance | 7p12.3 | 7 | 45697412 | 45697412 | p.Arg412His | 4.47 | 1.00 |
| FE23 | nonsynonymous SNV | OLFM1 | NA | Uncertain significance | 9q34.3 | 9 | 138011827 | 138011827 | p.Gly403Ser | 3.53 | 1.00 |
| FE24 | stopgain SNV | HSPA12A | NA | Uncertain significance | 10q25.3 | 10 | 118464681 | 118464681 | p.Glu96Ter | 2.22 | 0.99 |
| FE25 | stopgain SNV | FAT3 | NA | Uncertain significance | 11q14.3 | 11 | 92086959 | 92086959 | p.Glu561Ter | 1.68 | 1.00 |
| FE26 | stopgain SNV | RDX | NA | Uncertain significance | 11q22.3 | 11 | 110108333 | 110108333 | p.Arg379Ter | 0.91 | 0.96 |
| FE27 | stopgain SNV | KDM5A | NA | Uncertain significance | 12p13.33 | 12 | 416782 | 416782 | p.Trp1256Ter | 2.42 | 1.00 |
| FE28 | stopgain SNV | SLTM | NA | Uncertain significance | 15q22.1 | 15 | 59191864 | 59191864 | p.Gln288Ter | 2.71 | 1.00 |
| FE29 | stopgain SNV | NUP85 | NA | Uncertain significance | 17q25.1 | 17 | 73229175 | 73229175 | p.Arg513Ter | 0.85 | 1.00 |
| FE30 | stopgain SNV | PIP5K1C | NA | Uncertain significance | 19p13.3 | 19 | 3643341 | 3643341 | p.Glu517Ter | 2.28 | 0.97 |
| FE31 | nonsynonymous SNV | PPFIA3 | NA | Uncertain significance | 19q13.33 | 19 | 49637069 | 49637069 | p.Gly393Glu | 5.61 | 1.00 |
| FE32 | nonsynonymous SNV | KCNG1 | NA | Uncertain significance | 20q13.13 | 20 | 49620826 | 49620826 | p.Val431Ala | 4.34 | 0.42 |
| FE33 | stopgain SNV | AP1B1 | NA | Uncertain significance | 22q12.2 | 22 | 29724869 | 29724869 | p.Arg931Ter | 3.20 | 1.00 |
| FE34 | stopgain SNV | GSPT2 | NA | Uncertain significance | Xp11.22 | X | 51488460 | 51488460 | p.Gln580Ter | 2.54 | 0.99 |

Legend: ACMG-AMP: the American College of Medical Genetics and Genomics and the Association for Molecular Pathology, Chr: chromosome, GRCh37: Genome Reference Consortium Human Build 37, Mis_z: Z-score for missense intolerance of a gene, pLI: probability for the loss-of-function variant intolerance of a gene, Kb: kilo base pairs, CN: copy number.

**Supplementary Table 2: Pathogenic, likely pathogenic, and variants of uncertain significance in individuals with generalized epilepsy**

| **ID** | **Variant type** | **Gene/s** | **Associated neurological / psychiatric disorder** | **ACMG-AMP classification** | **CytoBand** | **Chr** | **Start_GRCh37** | **Stop_GRCh37** | **Consequence / nucleotide change** | **Mis_z** | **pLI** |
| --- | --- | --- | --- | --- | --- | --- | --- | --- | --- | --- | --- |
| GE1 | 2.67Mb Deletion | 12 genes | epilepsy | Pathogenic | 16p13.11-p12.3 | 16 | 15491006 | 18165043 | CN loss | - | - |
| GE2 | stopgain SNV | DSP | NA | Uncertain significance | 6p24.3 | 6 | 7581245 | 7581245 | p.Gln1608Ter | 0.89 | 1.00 |
| GE3 | splicing SNV | SMCHD1 | NA | Uncertain significance | 18p11.32 | 18 | 2739429 | 2739429 | c.3426-1G>A | 4.00 | 1.00 |
| GE4 | stopgain SNV | SAMD4B | NA | Uncertain significance | 19q13.2 | 19 | 39866481 | 39866481 | p.Gln287Ter | 3.03 | 1.00 |
| GE5 | nonsynonymous SNV | PPFIA3 | NA | Uncertain significance | 19q13.33 | 19 | 49651440 | 49651440 | p.Val979Ala | 5.61 | 1.00 |
| GE6 | stopgain SNV | GRAMD4 | NA | Uncertain significance | 22q13.31 | 22 | 47059981 | 47059981 | p.Trp228Ter | 2.59 | 1.00 |

Legend: ACMG-AMP: the American College of Medical Genetics and Genomics and the Association for Molecular Pathology, Chr: chromosome, GRCh37: Genome Reference Consortium Human Build 37, Mis_z: Z-score for missense intolerance of a gene, pLI: probability for the loss-of-function variant intolerance of a gene, Mb: mega base pairs, CN: copy number.

**Supplementary Table 3: Public variant databases**

| Variant databases | Individuals, N | Type |
| --- | --- | --- |
| GnomAD^1^ | 138,632 | WGS & WES |
| DiscovEHR^2^ | 50,726 | WES |
| HRCr1^3^ | 32,488 | WGS & WES |
| 2KJPN^4^ | 2,049 | WGS |
| GME^5^ | 1,794 | WES |
| ABraOM^6^ | 609 | WES |

Legend: N: number of individuals, WGS: whole-genome sequencing, WES: whole-exome sequencing.

**Supplementary Table 4: Percentage of females and males with PNES and comorbid disorders or a family history of seizures**

| **Comorbidities and family history** | **PNES** | | **χ2** | ***P*** |
| --- | --- | --- | --- | --- |
|  | **Females, N (%)** | **Males, N (%)** |  |  |
| Depression | 50 (65.8%) | 14 (53.8%) | 1.182 | 0.277 |
| Anxiety | 44 (57.9%) | 10 (38.5%) | 2.937 | 0.087 |
| Bipolar Disorder | 10 (13.2%) | 1 (3.8%) | 1.746 | 0.186 |
| PTSD | 12 (15.8%) | 2 (7.7%) | 1.073 | 0.3 |
| Chronic Pain | 14 (18.4%) | 4 (15.4%) | 0.123 | 0.726 |
| Family history of seizures | 70 (92.1%) | 24 (92.3%) | 0.001 | 0.974 |

Legend: N: number of individuals, χ2: test statistic, *P*: *P*-value.

**Supplementary Table 5: History of trauma or abuse and risk factors for epilepsy in the P/LP variant and VUS carriers with PNES**

| **ID** | **Identified variant (ACMG-AMP classification)** | **History of trauma or abuse** | **History of febrile seizures** | **History of head trauma/traumatic brain injury** | **History of CNS vascular disease** | **Other vascular comorbidities** | **History of CNS infection** | **History of CNS tumors** |
| --- | --- | --- | --- | --- | --- | --- | --- | --- |
| PNES1 | Pathogenic | No | No | No | No | No | No | No |
| PNES2 | Pathogenic | No | No | No | No | Yes | No | No |
| PNES3 | Pathogenic | Yes | No | Yes | No | No | No | No |
| PNES4 | Likely pathogenic | No | No | Yes | No | Yes | No | No |
| PNES5 | Pathogenic | Yes | No | No | No | No | No | No |
| PNES6 | Likely pathogenic | Yes | No | Yes | No | Yes | No | No |
| PNES7 | Uncertain significance | No | No | Yes | No | Yes | No | No |
| PNES8 | Uncertain significance | No | No | Yes | No | No | No | No |
| PNES9 | Uncertain significance | Yes | No | Yes | No | Yes | No | No |
| PNES10 | Uncertain significance | Yes | No | Yes | No | No | No | No |
| PNES11 | Uncertain significance | Yes | No | Yes | No | No | No | No |
| PNES12 | Uncertain significance | Unknown | No | Yes | No | No | No | No |

Legend: CNS: central nervous system.

**Supplementary Table 6: Summary statistics of the history of trauma or abuse and risk factors for epilepsy in all individuals with PNES**

|  | **History of trauma or abuse** | | | |
| --- | --- | --- | --- | --- |
| **Carrier status** | **Yes (%)** | **No** | **Unknown** | **Total** |
| P/LP variants | 3 (50%) | 3 (50%) | 0 | 6 |
| VUS | 3 (50%) | 2 (40%) | 1 (10%) | 6 |
| No variants identified | 30 (33.3%) | 25 (27.8%) | 35 (38.9%) | 90 |
|  |  |  |  |  |
|  | **Any risk factor for epilepsy*** | | | |
| **Carrier status** | **Yes** | **No** | **Unknown** | **Total** |
| P/LP | 3 (50%) | 3 (50%) | 0 | 6 |
| VUS | 6 (100%) | 0 | 0 | 6 |
| No variants identified | 55 (61.1%) | 35 (38.9%) | 0 | 90 |

Legend: P/LP: pathogenic or likely pathogenic, VUS: variants of uncertain significance.

* = combined history of febrile seizures, head trauma/traumatic brain injury, CNS vascular disease, other vascular comorbidities, CNS infection, or CNS tumors.

**2. Supplementary Figures**


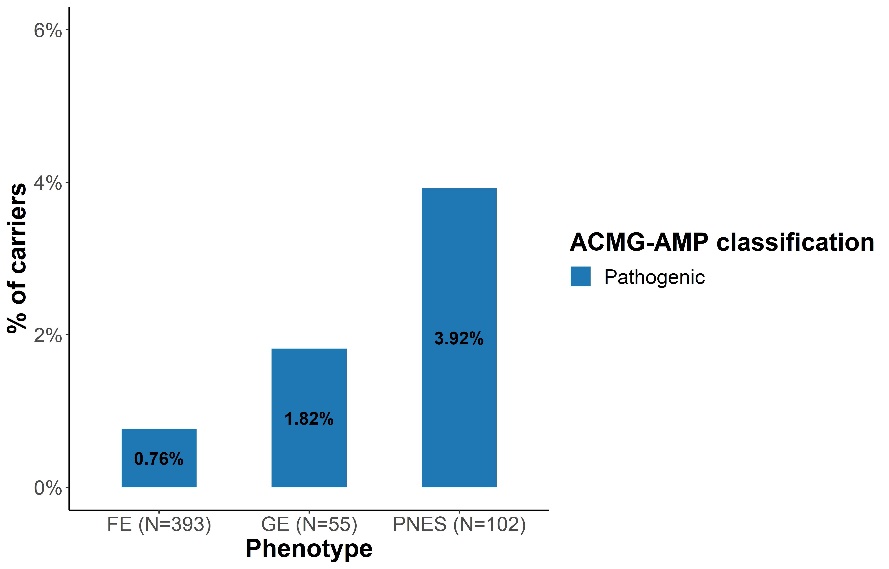


**Supplementary Figure 1: Burden of copy number variations (CNVs)**

Each bar plot represents the total percentage of carriers of pathogenic variants, highlighted in blue. There were no CNVs classified as likely pathogenic or of uncertain significance. The classification of the variants in the individuals with PNES was performed for research purposes only. Legend: FE: focal epilepsy, GE: generalized epilepsy, PNES: psychogenic nonepileptic seizures, N: number of individuals with each phenotype, ACMG-AMP: American College of Medical Genetics and Genomics and the Association for Molecular Pathology.


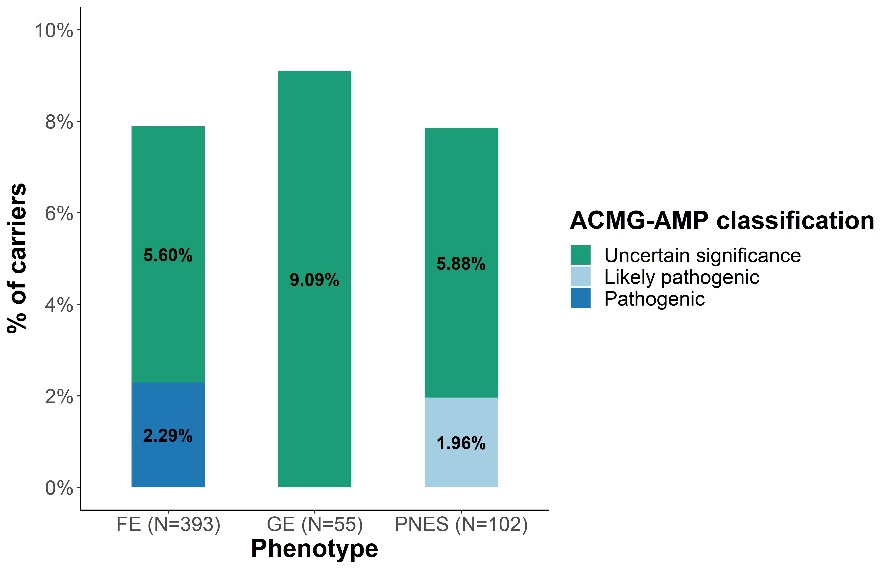


**Supplementary Figure 2: Burden of single nucleotide variants (SNVs)**

Each stacked bar plot represents the total percentage of carriers of i) pathogenic variants, highlighted in blue; ii) likely pathogenic variants, highlighted in light blue; and iii) variants of uncertain significance, highlighted in green. The classification of the variants in the individuals with PNES was performed for research purposes only. FE: focal epilepsy, GE: generalized epilepsy, PNES: psychogenic nonepileptic seizures, N: number of individuals with each phenotype, ACMG-AMP: American College of Medical Genetics and Genomics and the Association for Molecular Pathology.


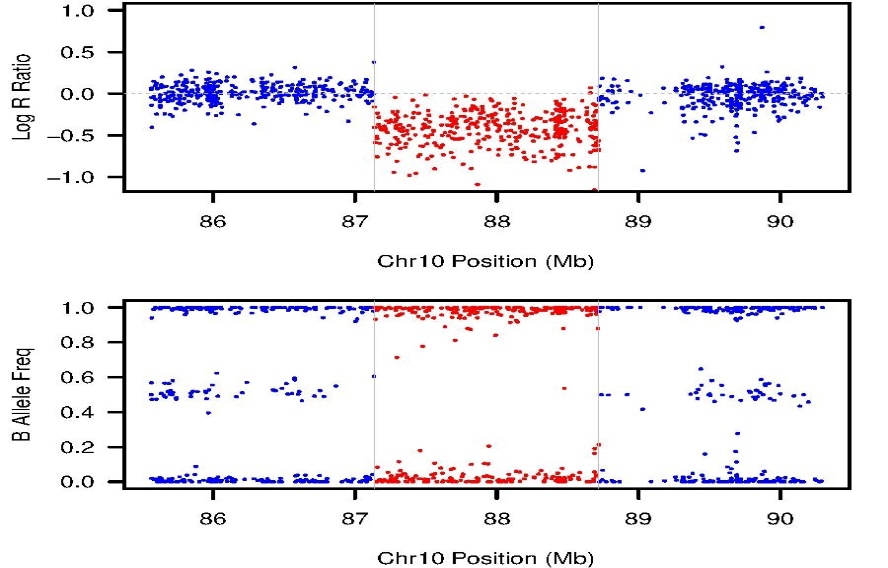


**Supplementary Figure 3: 10q23.1-q23.2 deletion identified in an individual with PNES (ID: PNES1)**

The upper plot is showing the signal intensity log R Ratio of each SNP (i.e., log2(observed intensity/reference intensity). The lower plot is showing B-allele frequency of each SNP (normalized measure of the allelic intensity ratio of two alleles A and B). The SNPs supporting the CNV are highlighted in red.


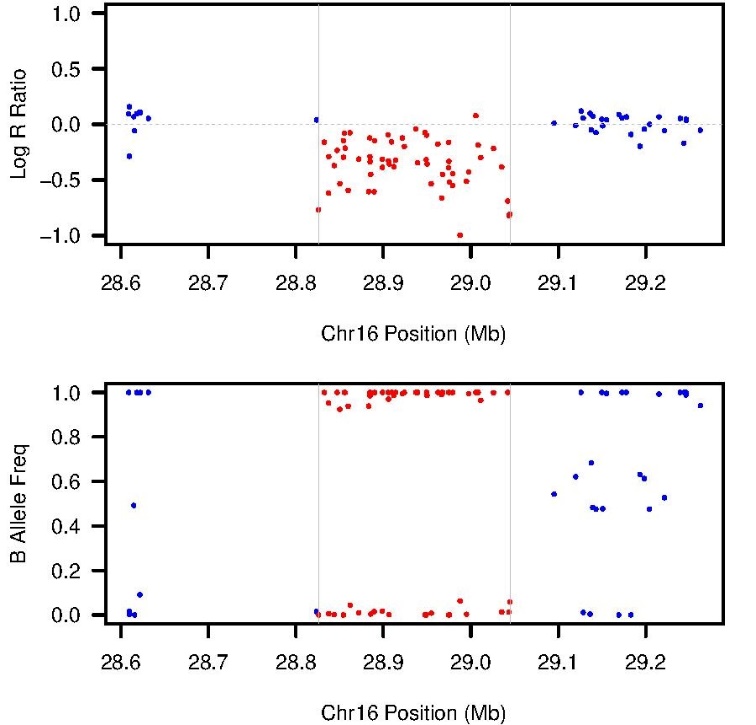


**Supplementary Figure 4: Distal 16p11.2 deletion identified in an individual with PNES (ID: PNES2)**

The upper plot is showing the signal intensity log R Ratio of each SNP (i.e., log2(observed intensity/reference intensity). The lower plot is showing B-allele frequency of each SNP (normalized measure of the allelic intensity ratio of two alleles A and B). The SNPs supporting the CNV are highlighted in red.


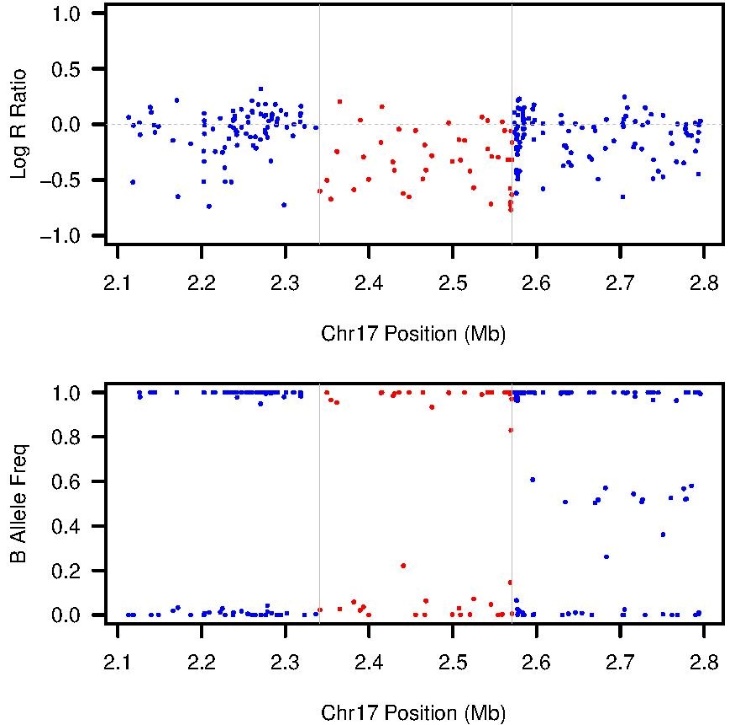


**Supplementary Figure 5: 17p13.3 deletion identified in an individual with PNES (ID: PNES3)**

The upper plot is showing the signal intensity log R Ratio of each SNP (i.e., log2(observed intensity/reference intensity). The lower plot is showing B-allele frequency of each SNP (normalized measure of the allelic intensity ratio of two alleles A and B). The SNPs supporting the CNV are highlighted in red.


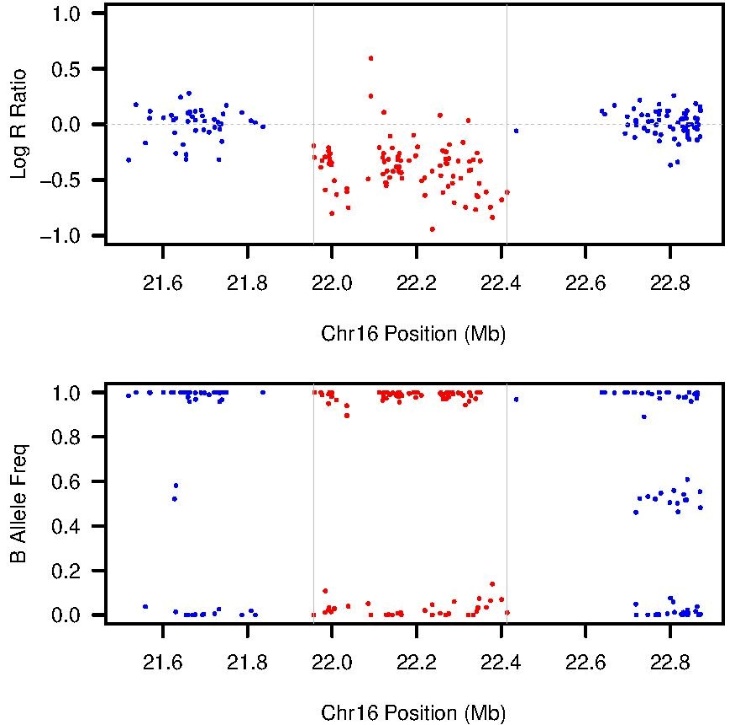


**Supplementary Figure 6: 16p12.2 deletion identified in an individual with FE (ID: FE4)**

The upper plot is showing the signal intensity log R Ratio of each SNP (i.e., log2(observed intensity/reference intensity). The lower plot is showing B-allele frequency of each SNP (normalized measure of the allelic intensity ratio of two alleles A and B). The SNPs supporting the CNV are highlighted in red.


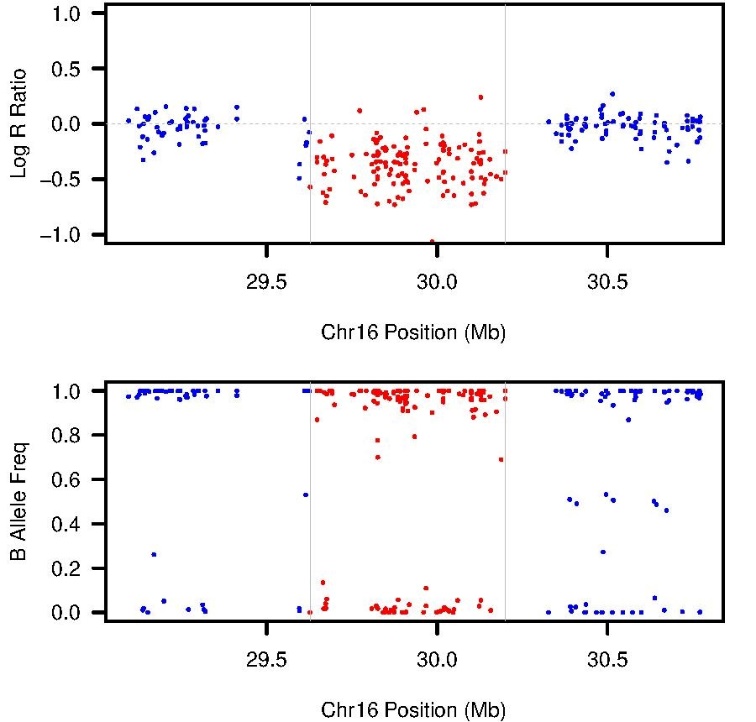


**Supplementary Figure 7: 16p11.2 deletion identified in an individual with FE (ID: FE5)**

The upper plot is showing the signal intensity log R Ratio of each SNP (i.e., log2(observed intensity/reference intensity). The lower plot is showing B-allele frequency of each SNP (normalized measure of the allelic intensity ratio of two alleles A and B). The SNPs supporting the CNV are highlighted in red.


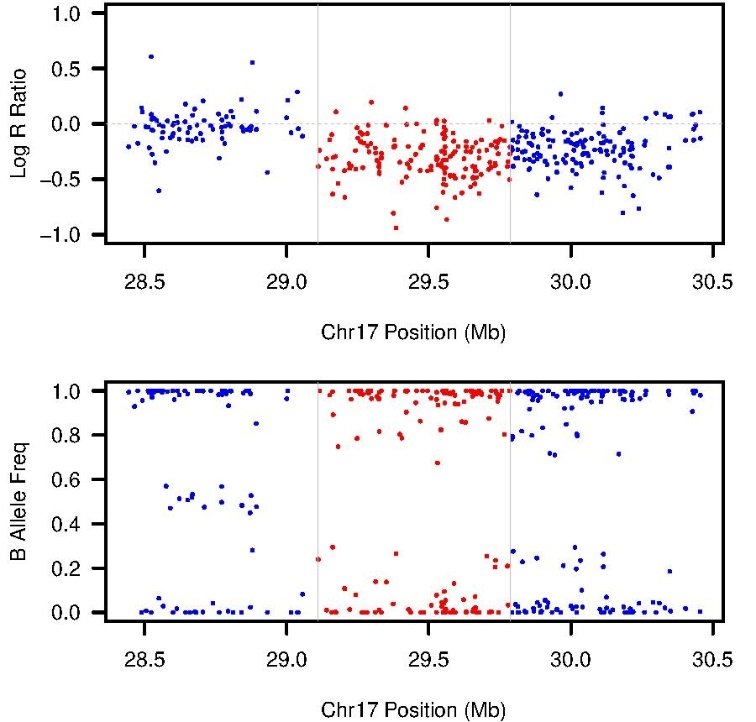


**Supplementary Figure 8: 17q11.2 deletion identified in an individual with FE (ID: FE6)**

The upper plot is showing the signal intensity log R Ratio of each SNP (i.e., log2(observed intensity/reference intensity). The lower plot is showing B-allele frequency of each SNP (normalized measure of the allelic intensity ratio of two alleles A and B). The SNPs supporting the CNV are highlighted in red.


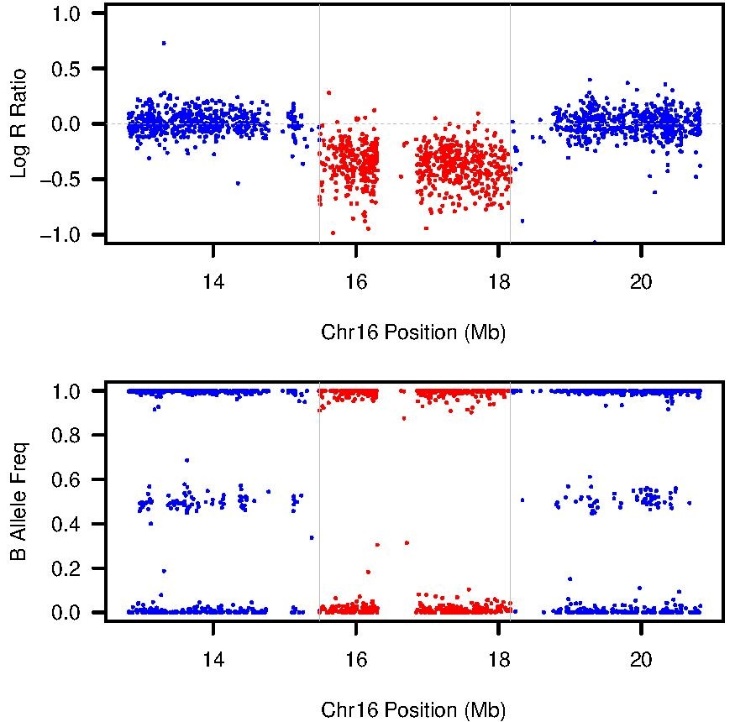


**Supplementary Figure 9: 16p13.11-p12.3 deletion identified in an individual with GE (ID: GE1)**

The upper plot is showing the signal intensity log R Ratio of each SNP (i.e., log2(observed intensity/reference intensity). The lower plot is showing B-allele frequency of each SNP (normalized measure of the allelic intensity ratio of two alleles A and B). The SNPs supporting the CNV are highlighted in red.

**3. References**

1 Karczewski KJ *et al.* Variation across 141,456 human exomes and genomes reveals the spectrum of loss-of-function intolerance across human protein-coding genes: Supplementary Information. *bioRxiv* 2019; published online Jan 30. DOI:10.1101/531210.

2 Dewey FE *et al.* Distribution and clinical impact of functional variants in 50,726 whole-exome sequences from the DiscovEHR study. *Science* 2016; **354**. DOI:10.1126/science.aaf6814.

3 McCarthy S *et al.* A reference panel of 64,976 haplotypes for genotype imputation. *Nat Genet* 2016; **48**: 1279–83.

4 Yamaguchi-Kabata Y *et al.* Evaluation of reported pathogenic variants and their frequencies in a Japanese population based on a whole-genome reference panel of 2049 individuals. *J Hum Genet* 2018; **63**: 213–30.

5 Scott EM *et al.* Characterization of Greater Middle Eastern genetic variation for enhanced disease gene discovery. *Nat Genet* 2016; **48**: 1071–6.

6 Naslavsky MS *et al.* Exomic variants of an elderly cohort of Brazilians in the ABraOM database. *Hum Mutat* 2017; **38**: 751–63.
